# Morning vaccination enhances antibody response over afternoon vaccination: A cluster-randomised trial

**DOI:** 10.1016/j.vaccine.2016.04.032

**Published:** 2016-05-23

**Authors:** Joanna E. Long, Mark T. Drayson, Angela E. Taylor, Kai M. Toellner, Janet M. Lord, Anna C. Phillips

**Affiliations:** a School of Sport, Exercise and Rehabilitation Sciences, University of Birmingham, Birmingham B15 2TT, UK; b Institute of Immunology and Immunotherapy, University of Birmingham, Birmingham B15 2TT, UK; c Institute of Inflammation and Ageing, University of Birmingham, Birmingham B15 2TT, UK; d Institute of Metabolism and Systems Research, University of Birmingham, Birmingham B15 2TT, UK

**Keywords:** Ageing, Antibodies, Cluster-randomised, Influenza vaccine, Time of day, Vaccination

## Abstract

**Objectives:**

Older adults are less able to produce a protective antibody response to vaccinations. One factor that contributes to this is immune ageing. Here we examined whether diurnal variations in immune responses might extend to the antibody response to vaccination.

**Design:**

We utilised a cluster-randomised trial design.

**Setting:**

24 General Practices (GPs) across the West Midlands, UK who were assigned to morning (9–11 am; 15 surgeries) or afternoon (3–5 pm; 9 surgeries) vaccination times for the annual UK influenza vaccination programme.

**Participants:**

276 adults (aged 65+ years and without a current infection or immune disorder or taking immunosuppressant medication).

**Interventions:**

Participants were vaccinated in the morning or afternoon between 2011 and 2013.

**Main outcome measures:**

The primary outcome was the change in antibody titres to the three vaccine influenza strains from pre-vaccination to one month post-vaccination. Secondary outcomes of serum cytokines and steroid hormone concentrations were analysed at baseline to identify relationships with antibody responses.

**Results:**

The increase in antibody levels due to vaccination differed between morning and afternoon administration; mean difference (95% CI) for H1N1 A-strain, 293.3 (30.97–555.66) *p* = .03, B-strain, 15.89 (3.42–28.36) *p* = .01, but not H3N2 A-strain, 47.0 (−52.43 to 146.46) *p* = .35; those vaccinated in the morning had a greater antibody response. Cytokines and steroid hormones were not related to antibody responses. No adverse events were reported.

**Conclusions:**

This simple manipulation in the timing of vaccine administration to favour morning vaccination may be beneficial for the influenza antibody response in older adults, with potential implications for vaccination strategies generally.

**Trial registration:**

This trial is registered with the ISRCTN (ISRCTN70898162).

## Highlights

• Early small studies provide mixed evidence for effects of time of vaccination on antibody response.
• This is the first large scale randomised trial of different times of vaccination.
• Morning vaccination enhances the antibody response to the influenza vaccine.
• This simple manipulation is cost neutral and may improve protection from influenza in older adults.

## 1 Introduction

The influenza vaccination is part of the seasonal vaccination programme carried out by General Practice (GP) surgeries across the UK and in many other countries, with patients aged 65+ years being the majority of recipients. Despite this, the influenza virus is responsible for 250,000–500,000 thousand deaths annually [1] and older adults are the highest proportion of the hospitalisations and influenza-related mortalities [2]. Although the contributing factors are varied, the age-related decline in immunity reduces the ability of older adults to produce adequate antibody responses following vaccination [3], [4], compromising the protection given against the influenza virus.

A number of interventions have sought to improve the antibody response to vaccination. For example, the addition of adjuvants to the vaccine preparation, but these can have adverse side effects [5]. More recently, behavioural interventions prior to vaccination have been used, such as aerobic exercise [6], [7], [8], with some success. However, such interventions may be impractical in a public health setting.

Recent developments in chronobiology have revealed that the response of the immune system to challenge varies significantly with the time of day [9], [10] and 56 of the top 100 best-selling drugs in the United States target the product of a circadian gene [11] suggesting that the timing of vaccinations may also influence antibody responses. Indeed, circadian variations in responses to antigen have been observed in mice [12], [13]. The scant previous research in humans has produced mixed results. An attenuated Venezualan equine encephalomyelitis vaccine administered at 8 am resulted in peak antibody titres 4 days earlier than the peak in those vaccinated at 8 pm [14]. However, a hepatitis B vaccine administered in the afternoon between 1 and 3 pm yielded a higher antibody response, compared to vaccination between 7.30 and 9 am [15]. More recently, a convenience sample of 164 men and women showed that men exhibited a higher antibody response when vaccinated in the morning [16]. However, this study was not randomised and used a relatively small mixed sample of young and elderly populations and hepatitis A and influenza vaccinations, respectively. It is possible that diurnal variations in immune cell responses and/or levels of hormones with immune modifying properties, such as cortisol or inflammatory cytokines, provide an advantageous period for vaccination responses to occur. Therefore, adjusting the timing of vaccination may be a simple, cost neutral and effective public health intervention to improve vaccination responses, particularly in older adults. However, it is possible that the best time of day for vaccination may be different for different vaccines, as they stimulate different types of immune response for protection, e.g. thymus-dependent versus thymus-independent responses.

### 1.1 Rationale for cluster design and hypothesis

The present cluster-randomised trial aimed to determine whether randomising GP surgeries to administering the influenza vaccination to older adults in the morning (9–11 am) or afternoon (3–5 pm) impacted upon the magnitude of antibody responses at four weeks post vaccination. Timings were chosen to represent the two extremes of routine morning versus afternoon clinics, in keeping with GP surgery opening hours for practicality of future application. A cluster design was chosen to fit the practicalities of organising GP surgery vaccination clinics. It was hypothesised that morning vaccination would be more beneficial for antibody responses than afternoon vaccination, at both individual and cluster level.

## 2 Methods

### 2.1 Participants and eligibility criteria

298 participants were recruited from 24 Primary Care General Practices within the West Midlands, UK, with 276 being eligible for full data analysis. Eligibility criteria to participate in the study were: ≥65 years old, taking no medication which could influence immune function e.g. immune-suppressants, no current acute infections and no current cancer, diabetes, chronic inflammatory disease or immune disorder. There were no eligibility criteria for clusters except being an NHS GP surgery within the West Midlands UK area willing to take part in the trial and be randomised to vaccinating participants in one of the two time slots.

### 2.2 Trial design

This was a non-blinded cluster-randomised trial. This study was approved by the South Birmingham Local Research Ethics committee and funded by an MRC Lifelong Health and Wellbeing Collaborative Research Grant. This trial is registered with the ISRCTN as a controlled trial (ISRCTN70898162). The protocol is available from the corresponding author or in the trial registry.^2^

### 2.3 Intervention

Participants were invited to take part in the study by a letter sent from their GP surgery on behalf of the research sponsor (University of Birmingham (UB)) and they returned the signed written informed consent form to the research team at UB. GP surgeries (clusters) where participants had returned consent forms were then notified of which arm of the trial (morning or afternoon) they had been randomised to. Participants were then invited to attend on two separate occasions, one month apart. The initial session involved providing a blood sample and receiving the trivalent influenza vaccination as standard practice (administered intra-muscularly) between either 9 and 11 am or 3 and 5 pm. In accordance with standard GP practise, the standard influenza vaccine used routinely during each influenza season was administered using the standard single dose (0.5 ml), route of administration (intramuscularly into deltoid) and common commercially available inactivated preparations in pre-filled syringes in 2011/12: Pfizer Ltd Enzira^®^ split virion or Sanofi Pasteur split virion; in 2012/13: Pfizer Ltd Enzira^®^ split virion, Sanofi Pasteur split virion, BGP Products Ltd Imuvac^®^ surface antigen or GlaxoSmithKline FLUARIX^®^ split virion; and in 2013/14: Pfizer Limited Enzira^®^ or generic split virion, Sanofi Pasteur split virion, BGP Products Ltd Imuvac^®^ surface antigen, GlaxoSmithKline FLUARIX^®^ split virion, or Janssen-Cilaq Viroflu^®^ surface antigen; the exact influenza components these contained are detailed below. A questionnaire pack was given to participants to complete at home and return by mail. One month later, participants returned to their GP practice to give a morning fasted blood sample and have weight, height and waist to hip ratio measurements taken. Number of previous influenza vaccinations the participant had received was gained from GP electronic records.

### 2.4 Questionnaires

Participants completed a battery of questionnaires at baseline to assess socio-demographics and health behaviours. Health behaviours (smoking, alcohol consumption and sleep duration) were assessed using a questionnaire adapted from the Whitehall II study [17]; smoking and drinking alcohol were dichotomised into yes/no variables.

### 2.5 Blood sampling and analysis

Blood was collected in to anti-coagulant free tubes (BD Vacutainer, UK) and clotted at room temperature before centrifugation at 2000 × *g* for 5 min. The separated serum was frozen at −20 °C for later analysis.

#### 2.5.1 Haemagglutination inhibition assay

Anti-influenza antibody titres were measured using an in-house haemagglutination inhibition test as described in the WHO Manual for Animal Influenza Diagnosis and Surveillance [18]. The 2011–2012 influenza vaccine contained viral strains: A/California/7/2009 (H1N1), A/Perth/16/2009 (H3N2) and B/Brisbane/60/2008 (B). The 2012–2013 influenza vaccine contained viral strains: A/California/7/2009 (H1N1), A/Victoria/361/2011 (H3N2) and B/Wisconsin/1/2010 (B) and the 2013–2014 influenza vaccine contained viral strains: A/California/7/2009 (H1N1), A/Texas/50/2012 (H3N2) and B/Massachusetts/2/2012 (B). Details of this assay method have been described elsewhere [19].

#### 2.5.2 Cytokine assay

Multiplex technology was used to assay serum cytokines IL-6 and IL-10 in duplicate according to the manufacturer's specifications (BioRad Laboratories, UK). Acquisition software (BioPlex Software Manager version 4, BioRad Laboratories, CA, USA) was used to generate cytokine concentrations from a five parameter logistic curve fit.

#### 2.5.3 Steroid analysis

Liquid chromatography tandem mass spectrometry was used for the analysis of seven steroids in serum (cortisol, cortisone, corticosterone, 11-deoxycortisol, testosterone, dehydroepiandrosterone (DHEA) and androstenedione). All steroids were extracted via liquid/liquid extraction, analysed, derivatised and re-analysed as previously described [20]. Quantification was achieved through reference to a calibration series which spans the expected concentration range of the analyte 0.25–500 ng/mL.

### 2.6 Outcomes

The primary outcome for this trial was the change in antibody titre from baseline pre-vaccination to one month post-vaccination at the individual level. Secondary outcome measures were cytokine and steroid hormone levels, as potential underlying mechanisms of any effect of time of day and/or gender on vaccination response.

### 2.7 Sample size

The initial sample size was determined on the basis of our previous study which found a mean difference in log10 antibody titre between morning and afternoon vaccination of 0.27 for men. However, this previous research was an opportunistic study and there is good evidence that the effect sizes in non-randomised studies are much larger than those typically found in randomised studies. Consequently, with a mean difference of 0.17, power at 0.90, alpha at 0.05, within and between cluster variance of 0.0985 and 0.0036, respectively, the number of men required in two groups of 8 surgeries would be 13 per surgery. This would give 104 men in each arm of the trial, 208 men in all from 16 surgeries. Likewise a separate comparison of females in the two arms would require 104 females in each arm making a total of 416 patients in all.

### 2.8 Randomisation and blinding

**Fig. 1:**
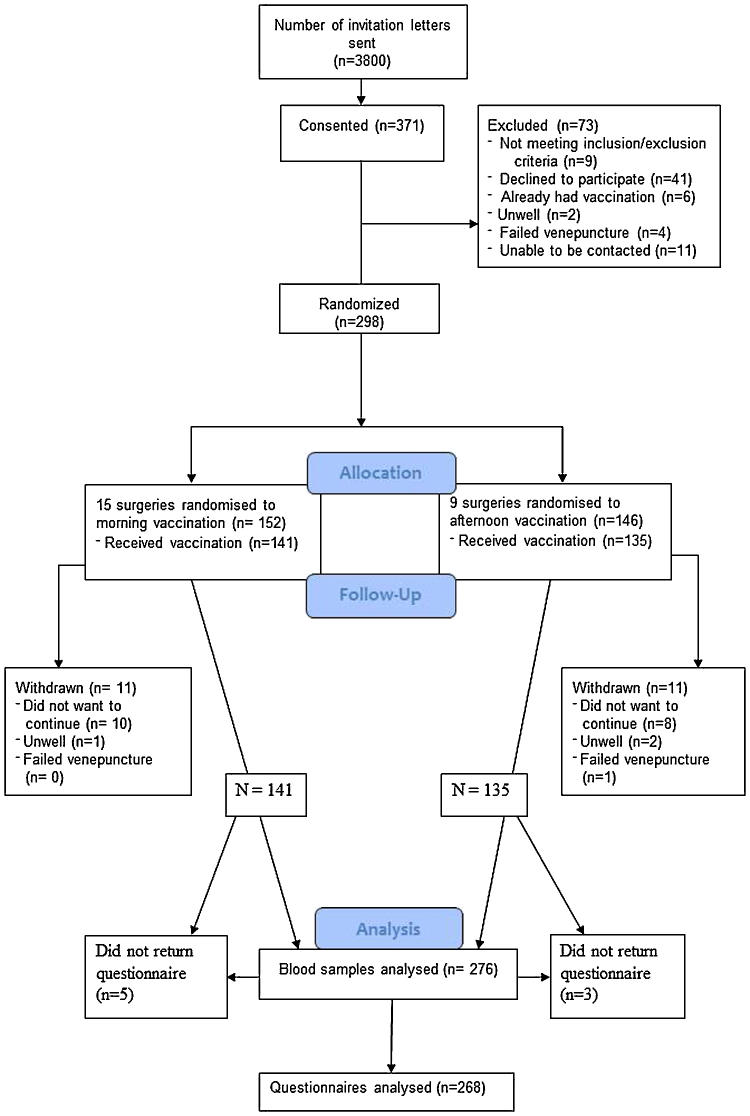
CONSORT diagram of participant recruitment and retention throughout the study.

General Practices who agreed to take part in the trial were cluster-randomised by the research team annually each influenza season through random selection of morning or afternoon documents from an opaque envelope, which were then assigned sequentially to the list of participating surgeries by JEL. This meant surgeries (clusters) were randomised to administer either a morning (9–11 am) (*N* = 141) or afternoon (3–5 pm) (*N* = 135) vaccination (see Fig. 1 for CONSORT diagram). As randomisation was annual it was possible for the same GP practice to be randomised to different arms in different years of the study. Due to the nature of randomising to different times of day, blinding was not possible.

### 2.9 Statistical analysis

Analyses were carried out using IBM SPSS version 21.0 (IBM SPSS Inc, Chicago, IL) by the lead author (AP). Differences between the intervention arms (morning versus afternoon) in baseline participant socio-demographic characteristics were analysed using one way analysis of variance (ANOVA) for continuous data or chi-squared test, as appropriate. As antibody titres were measured over three years, similar antibody strains within the influenza vaccination were combined over the three years in order to examine responses to H1N1, H3N2, and the B strains overall in each vaccine; these yielded three new variables, H1N1 combined (all A/California strains), H3N2 combined (2011 A/Perth, 2012 A/Victoria and 2013 A/Texas) and B combined (2011 B/Brisbane, 2012 B/Wisconsin and 2013 B/Massachusetts). This method of combining influenza strains has been used previously [21]. Mixed modelling was used to examine the effect of the intervention with one-month antibody titre as the dependent variable, baseline antibody titre as a covariate, and, due to cluster-randomisation, GP surgery as a random effect.

Given our previous findings with regard to time of day x sex interaction effects [16], separate exploratory covariate analyses were run, entering time of day × sex as an interaction term. To determine whether any intervention effects were mediated by cytokines or steroid hormone levels further separate exploratory models were run with each cytokine or hormone as an additional covariate.

Missing data for antibody titre led to the exclusion of participants, but missing data for exploratory covariates were minimal, thus analyses were run on the available data excluding participants without complete data; variations in degrees of freedom reflect this.

## 3 Results

### 3.1 Participant characteristics

Participants were enrolled between 28th October 2011 and 12th November 2013 and participant flow is shown in the CONSORT diagram (Fig. 1). 298 participants gave informed consent and 15 and 9 GP surgeries were randomised to morning and afternoon vaccination administration, respectively, and 276 participants provided full data on the primary outcomes. The final follow-up appointment was conducted in December 2013. There were no serious adverse effects reported for this trial. Table 1 shows baseline socio-demographics and health behaviours between morning and afternoon vaccination clusters; there were no significant differences between groups.

### 3.2 Associations between time of day of vaccination and antibody response

Repeated measures ANOVA showed that all combined strains had a significant main effect of time, such that there was an increase in antibody levels between baseline and one month (H1N1 combined (*p* = .001), H3N2 combined (*p* < .001), and B combined (*p* < .001)).

**Fig. 2:**
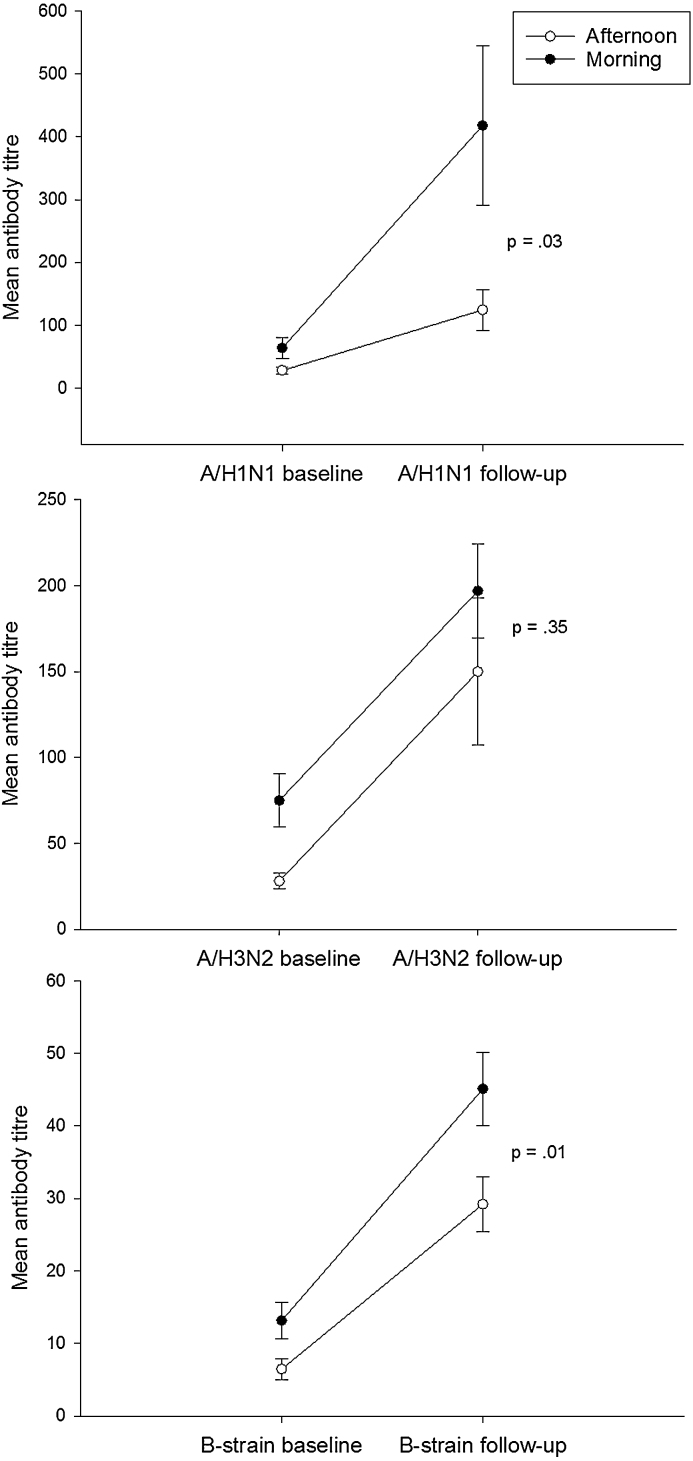
Antibody titres following vaccination for each influenza strain; A/H1N1, A/H3N2 and B. Antibody levels were determined by haemagglutination inhibition and the values shown are the 1 month post vaccination data adjusted for the baseline values. Data are mean ± SE (*N* = 276) and the *p* value indicates a difference between the morning and afternoon.

Fig. 2 shows mean (SE) data for antibody titres at baseline and follow-up. There were significant effects of time of day for the A/H1N1 strain (*p* = .03) and the B strain (*p* = .01) but not for the A/H3N2 strain (*p* = .35), such that morning vaccination resulted in a greater antibody response. Mean differences (95% CI) were 293.3 (30.97–555.66) for A/H1N1, 47.0 (−52.43 to 146.46) for A/H3N2, and 15.89 (3.42 to 28.36) for the B strain. There were no significant time of day × sex interactions.

### 3.3 Sensitivity analysis

As different vaccine preparations were used by the different GP surgeries, we repeated the above analysis adjusting for vaccine type as a covariate. This did not change the findings (*p* = .03, .35, and .01 as before).

### 3.4 Associations between time of day of vaccination and systemic biomarkers

Table 2 shows the cytokine and steroid hormone data for the two groups; as expected, there were significant differences between groups for cortisol, cortisol:cortisone ratio, corticosterone, DHEA and androstenedione. To examine whether any of these biomarkers mediated the association between time of day and vaccination response, each significant biomarker was entered singly into the mixed models as a covariate. If the differences between the trial arms became non-significant upon entry of each covariate, this would indicate that the time of day effects were mediated, at least in part, by the biomarker under examination, and suggest that a formal test of mediation should be run. In models examining antibody titre, covariate adjustment for these five steroids did not change the previous findings; *p* = .03, .35, and .01 for the A/H1N11, A/H3N2, and B strains, respectively. These models were rerun with all of the covariates added simultaneously to examine whether there might be an effect of multiple covariates, the results again remained largely unchanged, *p* = .03, .37, and .02 for the A/H1N11, A/H3N2, and B strains, respectively, indicating no evidence of mediation by these covariates.

## 4 Discussion

This study examined whether manipulating the time of day an older adult received their annual influenza vaccination would have an effect on the magnitude of the antibody response at one month. Results showed that antibody responses to two of three influenza strains were higher when the vaccination was given in the morning. Whether these differences relate to clinical disease resistance remains to be examined. Further, although several steroid hormones and cytokines differed between the trial arms, as expected given their known diurnal rhythms, covariate adjustment did not reveal any mediation of the time of day effect by these factors.

These findings show some similarities to our previous pilot study [16], where men vaccinated in the morning had a greater antibody response to hepatitis A and the A/Panama influenza strain. However, the present study has greater power (276 versus 164 participants), was a cluster randomised trial and used only older adults, rather than a mix of young and old subjects. Further, there were no interactions between time of vaccination and sex in the present study such that both men and women showed greater responses in the morning. It is not clear why we did not find a sex difference in the present analysis, but it fits with the lack of evidence of sex differences in diurnal rhythms [22], [23].

With regard to steroid hormones as a potential mechanism of effect, higher levels of cortisol, cortisol:cortisone and corticosterone, and lower levels of DHEA and androstenedione, were observed among those vaccinated in the morning. This is expected due to the diurnal rhythm of cortisol, with levels peaking 30 min post awakening then decreasing throughout the day [24]. Although higher levels of cortisol are generally associated with immune suppression, cortisol is immunoregulatory and can boost immunity [25], and higher levels have previously been associated with greater antibody responses, in the context of acute psychological stress [26], [27]. However, neither cortisol nor the other hormones measured mediated the effects observed here.

### 4.1 Strengths and limitations

The present study was the first large scale randomised trial of different times of vaccination and provides evidence that morning vaccination enhances the antibody response to the influenza vaccine. However, the trial has some limitations. First, we were not able to reach our target recruitment of 400 participants over three years, and additional power might have made our indicative results stronger. Second, due to the smaller numbers and different influenza strains in the vaccine across the three years of the study, we were forced to combine responses across similar strains for analysis. However, this method has been used previously in similar research [21] and provides additional confidence that our findings are generalisable beyond one year of influenza vaccine strains. Third, as the present study aimed to fit in with usual practice at each GP surgery, it did not constrain each surgery to the choice of influenza vaccine used, thus each used their usual vaccine manufacturer, which differed between surgeries. However, although these varied, they contained the same influenza strains each year and work in the same manner with the same dose. Further, adjustment for the exact preparation used did not alter our main findings.

Our future research will focus on examining generalisability of these findings to different years, patient samples, and potentially different vaccinations. As suggested earlier, it is possible that the best time of day for vaccination may be different for different vaccines, as they stimulate different types of immune response for protection, e.g. thymus-dependent versus thymus-independent responses. These different types of response have been shown to be differentially susceptible to behavioural factors such as stress [28], thus it remains to be seen whether the different types of vaccine would also be differentially susceptible to the impact of the timing of vaccination. Further, it remains to be tested whether these differences in antibody titres observed here relate to clinical disease resistance.

### 4.2 Conclusions and implications for practice

In conclusion, this study provides preliminary evidence that a simple and cost neutral manipulation of the timing of vaccine administration may improve protection from the influenza virus if older adults are vaccinated in the morning.

## Authors’ contribution

MD conceived of the original idea and contributed to the final draft of the manuscript. JML, AP, and KMT developed the idea and gained research funding and contributed to drafting the manuscript. AP was Chief Investigator for the project. MD and JML oversaw laboratory assays. JEL and AT carried out the laboratory analyses. JEL collected all data and wrote the first draft of the manuscript. AP conducted the statistical analysis with input from a medical statistician, and finalised the manuscript. All authors contributed to the final manuscript. AP is the corresponding author and guarantor. All authors have had full access to the data and can take responsibility for the integrity of the data and accuracy of the data analysis.

## Funding

The sponsor was the University of Birmingham. The study was funded by an MRC Lifelong Health and Wellbeing Collaborative Research Grant. The funders and sponsor had no involvement in the design, collection, analysis, interpretation or writing up of the data, or the decision to submit.

## Conflicts of interest

The authors have no conflicts of interest. All authors have completed the Unified Competing Interest form at www.icmje.org/coi_disclosure.pdf (available on request from the corresponding author) and declare: support from the Medical Research Council (MRC) Lifelong Health and Wellbeing Collaborative Research Grant (G1001390) for this work. There are no other relationships or activities that could appear to have influenced the submitted work.

## Figures and Tables

**Table 1:** Mean (SD) descriptive variables for participants vaccinated in the morning and afternoon.

	Mean (SD)/*N* (%)		
	*N*	Morning	Afternoon
Number of GP surgeries	276	15	9
Number of participants	276	141	135
Age (years)	266	71.1 (5.42)	71.4 (5.51)
Number of previous vaccinations	216	5.1 (5.20)	5.0 (4.85)
BMI (kg/m^2^)	267	27.6 (4.75)	27.4 (4.16)
Waist-hip ratio	256	0.9 (0.12)	0.90 (0.08)
Exercise score	250	4.0 (3.26)	4.1 (3.33)
Sex (Female)	276	75 (53%)	61 (45.7%)
Ethnicity (White versus Asian/Black)	256	123 (73%)	128 (100%)
Occupation (non-manual)	241	52 (43%)	55 (46%)
Married (yes)	256	81 (63%)	88 (63%)
Taking medication (yes)	254	94 (74%)	93 (73%)
Smoking status (current smoker)	255	4 (3%)	10 (8%)
Drinks alcohol (yes)	252	110 (87%)	111 (88%)
Sleep (8+ h per night vs. 7 or less)	250	28 (23%)	32 (25%)

**Table 2:** Serum cytokines and steroids measured at the two vaccination times.

	Mean (SD)			
	*N*	Morning	Afternoon	*p*
IL-6 (pg/ml)	272	4.6 (14.32)	12.5 (59.65)	.13
IL-10 (pg/ml)	275	5.3 (19.19)	4.5 (17.24)	.68
Cortisol (nmol/L)	274	212.3 (94.64)	142.6 (84.38)	<.001
Cortisone (nmol/L)	274	40.2 (13.24)	37.1 (22.17)	.07
Cortisol:Cortisone	274	5.5 (2.20)	4.0 (1.36)	<.001
Corticosterone (nmol/L)	274	6.5 (9.42)	3.2 (4.85)	<.001
11-Deoxycortisol (nmol/L)	274	3.9 (6.41)	3.0 (4.83)	.17
DHEA (nmol/L)	268	4.1 (4.70)	5.4 (5.52)	.04
Testosterone (nmol/L)	268	6.4 (7.24)	5.4 (5.52)	.24
Androstenedione (nmol/L)	268	5.1 (3.42)	6.2 (3.74)	.01

## Footnotes

2 Following completion of the trial in 2013, the sponsor (University of Birmingham) through a query to the MHRA, reclassified this trial as a Clinical Trial of an Investigational Medicinal Product (CTIMP). The study was retrospectively submitted to an ethics committee appropriate for a CTIMP, Haydock NRES Committee North West, and received a favourable opinion through special ethics review. Following consideration by the sponsor and MHRA, it was deemed not justifiable to prevent the use of the trial data given that patients were to receive this medication regardless of whether this was done as part of the trial or as per routine practice. There were no adverse events reported, and the data and analyses were deemed reliable and appropriate by the Primary Care Clinical Trials Unit statistician at the University of Birmingham.
