# Corrigendum to ‘Morning vaccination enhances antibody response over afternoon vaccination: A cluster-randomised trial’ [Vaccine 34 (2016) 2679–2685]

**DOI:** 10.1016/j.vaccine.2016.08.031

**Published:** 2016-09-14

**Authors:** Joanna E. Long, Mark T. Drayson, Angela E. Taylor, Kai M. Toellner, Janet M. Lord, Anna C. Phillips

**Affiliations:** a School of Sport, Exercise and Rehabilitation Sciences, University of Birmingham, Birmingham B15 2TT, UK; b Institute of Immunology and Immunotherapy, University of Birmingham, Birmingham B15 2TT, UK; c Institute of Inflammation and Ageing, University of Birmingham, Birmingham B15 2TT, UK; d Institute of Metabolism and Systems Research, University of Birmingham, Birmingham B15 2TT, UK

Following our publication, we have engaged further methods of mixed-method cluster analysis and wish to present these to the reader. Analyses of the raw data (as in Fig. 2 of the paper) with baseline as a fixed factor reveal the following mean differences (95% CI) for H1N1 A-strain, 263.6 (−1.62 to 525.59) p = .05, H3N2 A-strain, 3.35 (−99.10 to 92.41) p = .95, and B-strain, 9.39 (−20.23 to 1.44) p = .09. Further, using log transformed data, the analogous statistics are: log mean difference (95% CI) for H1N1 A-strain, 0.53 (−1.00 to −0.07) p = .04, H3N2 A-strain, 0.20 (−0.08 to 0.48) p = .16, and B-strain, 0.23 (−0.49 to 0.03) p = .08. These reanalyses yield the same message as the original paper, but the effect for the B-strain now becomes non-significant/a trend.

The authors would like to apologise for any inconvenience caused.

